# Caught in a downward spiral? The bidirectional relationship between financial insecurity and mental health: a NESDA study

**DOI:** 10.1192/j.eurpsy.2026.10459

**Published:** 2026-07-31

**Authors:** A. N. Nelissen, N. J. de Bles, W. A. van Eeden, S. E. Bauduin, W. J. Maas, B. W. Penninx, E. J. Giltay

**Affiliations:** 1 Leiden University Medical Centre, Leiden; 2 Universitair Medisch Centrum Groningen, Groningen; 3 Amsterdam Universitair Medisch Centrum, Amsterdam, Netherlands

## Abstract

**Introduction:**

The relationship between financial insecurity and mental health is complex and likely bidirectional. Most studies on this topic have predominately examined these effects unidirectional and cross-sectionally.

**Objectives:**

Our study aims to investigate the longitudinal bidirectional relationship between depression and/or anxiety and financial insecurity, offering a deeper insight into the temporal relationships.

**Methods:**

Financial insecurity was assessed using two items on food insecurity and financial strain, rated on 3- and 4-point ordinal scales, respectively, measured at up to 4 timepoints during a 13-year follow-up of the NESDA cohort. Clinical diagnoses of depression and anxiety using the CIDI interview and symptom severity through the IDS and BAI scales were also assessed at each wave. Lagged multilevel models examined whether financial insecurity at baseline predicted changes in depression and anxiety and vice versa. All models adjusted for educational level, household contribute, age, and gender, as well as for the prior value of the outcome variable.

**Results:**

The 2,567 individuals with complete data were aged mean 42.4 years (SD 13.2), and 34.6% were female. Both higher food insecurity and financial strain significantly predicted increased odds of onset of depressive disorders (OR = 2.12, 95% CI: 1.43–3.15 and OR = 1.92, 95% CI: 1.51–2.45) (Figure 1), as well as greater depressive symptom severity at 13-year follow-up (p for trend < .001 and p = .001, respectively) (Figure 2). Associations with anxiety disorders and symptom severity at follow-up were also significant (Figure 1 & 2). Conversely, baseline depression and anxiety disorders were associated with increased financial insecurity at subsequent follow-up waves (Figure 3). Depressive symptoms were also a significant predictor of financial insecurity, whereas anxiety symptoms were not (Figure 3).

**Image 1:**
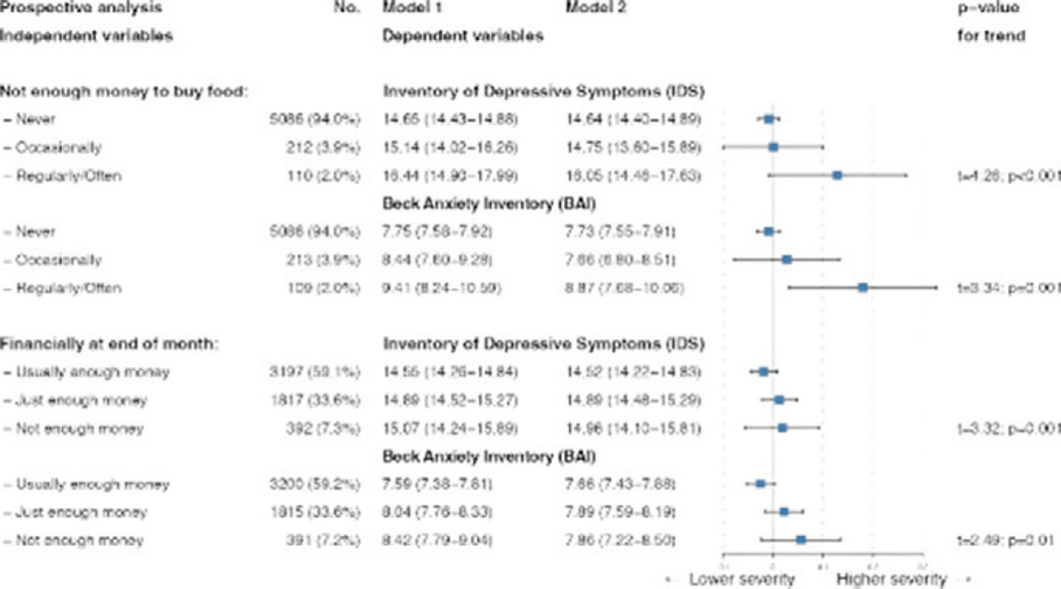
Image 1: Long description.

**Image 2:**
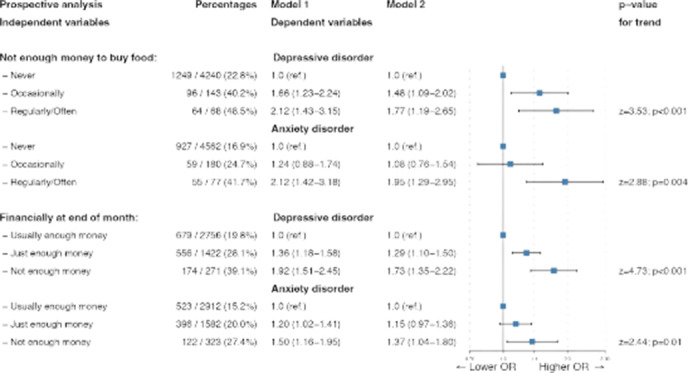
Image 2: Long description.

**Image 3:**
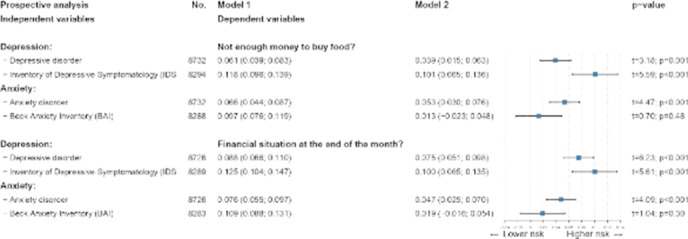
Image 3: Long description.

**Conclusions:**

Our findings support a bidirectional temporal association between financial strain and depression and anxiety.

**Disclosure of Interest:**

None Declared

## Long descriptions

### Image 1: Long description

The table is divided into two main independent variable sections.

1. Not enough money to buy food.

- Inventory of Depressive Symptoms I D S. Never: n=5086, Model 1: 14.65, Model 2: 14.64. Occasionally: n=212, Model 1: 15.14, Model 2: 14.75. Regularly/Often: n=110, Model 1: 16.44, Model 2: 16.05. P-value for trend: t=1.26, p < 0.001.

- Beck Anxiety Inventory B A I. Never: n=5086, Model 1: 7.75, Model 2: 7.73. Occasionally: n=213, Model 1: 8.44, Model 2: 7.66. Regularly/Often: n=109, Model 1: 9.41, Model 2: 8.87. P-value for trend: t=3.34, p < 0.001.

2. Financially at end of month.

- Inventory of Depressive Symptoms I D S. Usually enough money: n=3197, Model 1: 14.55, Model 2: 14.52. Just enough money: n=1817, Model 1: 14.89, Model 2: 14.89. Not enough money: n=392, Model 1: 15.07, Model 2: 14.96. P-value for trend: t=3.32, p < 0.001.

- Beck Anxiety Inventory B A I. Usually enough money: n=3200, Model 1: 7.59, Model 2: 7.66. Just enough money: n=1815, Model 1: 8.04, Model 2: 7.89. Not enough money: n=391, Model 1: 8.42, Model 2: 7.86. P-value for trend: t=2.49, p < 0.01.

The forest plot on the right visualizes Model 2 results, showing a rightward shift toward higher severity as financial hardship increases across all categories.

Navigate back to Image 1:.

### Image 2: Long description

The table is organized into two main independent variable sections.

1. Not enough money to buy food.

- For Depressive disorder, percentages increase from Never (22.8%) to Regularly/Often (48.5%). Model 1 O R increases from 1.0 (ref) to 2.12. Model 2 O R increases from 1.0 (ref) to 1.77.

- For Anxiety disorder, percentages increase from Never (16.9%) to Regularly/Often (41.7%). Model 1 O R increases from 1.0 (ref) to 2.12. Model 2 O R increases from 1.0 (ref) to 1.95.

- P-value for trend is less than 0.001 for depression and 0.004 for anxiety.

2. Financially at end of month.

- For Depressive disorder, percentages increase from Usually enough money (19.8%) to Not enough money (39.1%). Model 1 O R increases from 1.0 (ref) to 1.92. Model 2 O R increases from 1.0 (ref) to 1.73.

- For Anxiety disorder, percentages increase from Usually enough money (15.2%) to Not enough money (27.4%). Model 1 O R increases from 1.0 (ref) to 1.50. Model 2 O R increases from 1.0 (ref) to 1.37.

- P-value for trend is less than 0.001 for depression and 0.01 for anxiety.

The forest plot on the right visualizes Model 2 O R values with 95% confidence intervals. A vertical reference line is set at 1.0. All data points for ‘Occasionally’, ‘Regularly/Often’, ‘Just enough money’, and ‘Not enough money’ are positioned to the right of the reference line, indicating higher O R. The x-axis is labeled Lower O R to the left and Higher O R to the right.

Navigate back to Image 2:.

### Image 3: Long description

The table is divided into two main horizontal sections based on dependent variables.

Top section: Dependent variable is Not enough money to buy food?

1. Depressive disorder (N = 8732): Model 1 is 0.061; Model 2 is 0.039; t = 3.18, p < 0.001.

2. Inventory of Depressive Symptomatology I D S (N = 8294): Model 1 is 0.118; Model 2 is 0.101; t = 5.59, p < 0.001.

3. Anxiety disorder (N = 8732): Model 1 is 0.066; Model 2 is 0.053; t = 4.47, p < 0.001.

4. Beck Anxiety Inventory B A I (N = 8288): Model 1 is 0.097; Model 2 is 0.013; t = 0.70, p = 0.48.

Bottom section: Dependent variable is Financial situation at the end of the month?

1. Depressive disorder (N = 8728): Model 1 is 0.088; Model 2 is 0.075; t = 6.23, p < 0.001.

2. Inventory of Depressive Symptomatology I D S (N = 8289): Model 1 is 0.125; Model 2 is 0.100; t = 5.61, p < 0.001.

3. Anxiety disorder (N = 8726): Model 1 is 0.076; Model 2 is 0.047; t = 4.09, p < 0.001.

4. Beck Anxiety Inventory B A I (N = 8283): Model 1 is 0.109; Model 2 is 0.019; t = 1.04, p = 0.30.

The forest plot on the right visualizes Model 2 coefficients with 95 percent confidence intervals. Points to the right of the zero line indicate higher risk, while points to the left indicate lower risk. Most variables show a significant positive correlation with higher risk, except for the B A I measures which cross the zero line.

Navigate back to Image 3:.

